# The Global Impact of COVID-19 on Childhood Cancer Outcomes and Care Delivery - A Systematic Review

**DOI:** 10.3389/fonc.2022.869752

**Published:** 2022-04-07

**Authors:** Amna Majeed, Tom Wright, Biqi Guo, Ramandeep S. Arora, Catherine G. Lam, Alexandra L. Martiniuk

**Affiliations:** ^1^ Temerty Faculty of Medicine, University of Toronto, Toronto, ON, Canada; ^2^ School of Public Health, Faculty of Medicine and Health, The University of Sydney, Sydney, NSW, Australia; ^3^ Department of Medical Oncology, Max Super-Specialty Hospital, New Delhi, India; ^4^ Department of Global Pediatric Medicine and Oncology, St. Jude Children's Research Hospital, Memphis, TN, United States

**Keywords:** pediatric oncology, COVID-19, healthcare delivery, global health, pediatric clinical outcomes, LMIC, health systems and services

## Abstract

**Background:**

Childhood cancer represents a leading cause of death and disease burden in high income countries (HICs) and low-and-middle income countries (LMICs). It is postulated that the current COVID-19 pandemic has hampered global development of pediatric oncology care programs. This systematic review aimed to comprehensively review the global impact of COVID-19 on childhood cancer clinical outcomes and care delivery.

**Methods:**

A systematic search was conducted on PubMed, Embase, Medline, and the African Medical Index from inception to November 3, 2021 following PRISMA guidelines. A manual search was performed to identify additional relevant studies. Articles were selected based on predetermined eligibility criteria.

**Findings:**

The majority of studies reported patients with cancer and COVID-19 presenting as asymptomatic (HICs: 33.7%, LMICs: 22.0%) or with primary manifestations of fever (HICs: 36.1%, LMICs: 51.4%) and respiratory symptoms (HICs: 29.6%, LMICs: 11.7%). LMICs also reported a high frequency of patients presenting with cough (23.6%) and gastrointestinal symptoms (10.6%). The majority of patients were generally noted to have a good prognosis; however the crude mortality rate was higher in LMICs when compared to HICs (8.0% vs 1.8%). Moreover, the pandemic has resulted in delays and interruptions to cancer therapies and delays in childhood cancer diagnoses in both HICs and LMICs. However, these findings were disproportionately reported in LMICs, with significant staff shortages, supply chain disruptions, and limited access to cancer therapies for patients.

**Conclusions:**

The COVID-19 pandemic has resulted in delays and interruptions to childhood cancer therapies and delays in childhood cancer diagnoses, and disproportionately so within LMICs. This review provides lessons learned for future system-wide disruptions to care, as well as provides key points for moving forward better with care through the remainder of this pandemic.

**Systematic Review Registration:**

CRD42021266758, https://www.crd.york.ac.uk/prospero/display_record.php?RecordID=266758

## 1 Background

With over 400,000 children and adolescents developing cancer each year globally, cancer represents a leading cause of death and disease burden in pediatric populations (1). Moreover, pediatric oncology patients in low-and middle-income countries (LMICs) are five times as likely to die from a diagnosis of cancer compared to those in high-income countries, due to delays in diagnosis, obstacles to accessing care, and higher rates of relapse, amongst other reasons (2). In recognition of these disparities in cancer care delivery and outcomes, in 2018, the World Health Organization (WHO) launched the Global Initiative for Childhood Cancer with the support of St. Jude and other global partners, with the explicit aim to support childhood cancer programs, reduce global suffering, and increase survival including for six common childhood cancers to at least 60% for all by 2030 (3). Progress in this direction is likely to have been hampered by the COVID-19 pandemic, moving us further away from achieving this goal within the next decade (4).

As of January 16, 2022, there have been over 326 million cases and 5.54 million deaths from COVID-19, caused by the severe acute respiratory syndrome coronavirus 2 (SARS-CoV-2) and straining hospitals and healthcare systems worldwide (5). The pandemic has also impacted pediatric oncology patients. In contrast to adults, the disease has been shown to impact children to a milder degree with varying estimates (6). Additionally, with the emergence of new variants, these risk estimates in healthy children and in children with underlying risk factors are constantly changing (6). Indeed, in a large international sample of pediatric oncology patients with COVID-19, the Global Registry of COVID-19 in Childhood Cancer (GRCCC) created by St. Jude Global and the International Society of Pediatric Oncology reported that deaths attributed to the infection were approximately 4% (data collected as of February 2021) (7). This statistic is lower than the 13% of deaths reported in an analogous registry for adults with cancer (i.e., the COVID-19 and Cancer Consortium), but significantly higher than that of the general pediatric population (6–10).

Moreover, how COVID-19 is affecting cancer care delivery in pediatric populations in areas that have been severely impacted by COVID-19 is only starting to be understood. Pediatric oncology care is heavily reliant on prompt evaluation and diagnosis, multidisciplinary subspecialized teams, timely and coordinated therapy, and access to supportive care, amongst other measures (11–14). There exist inadequacies to accessing quality pediatric cancer care in many countries; the pandemic has only served to further exacerbate these disparities (14–16). Indeed, in a global study of 54 countries across six continents, Jazeih et al. (2020) reported challenges to adult cancer care due to COVID-19 (in April/May 2020). These challenges included overwhelmed healthcare systems, lack of personal protective equipment (PPE), staff shortages, and restricted access to medications which affected cancer care (17). These experiences are similar in the pediatric cancer care setting (12). COVID-19 has, and will, impede vital progress made towards the WHO Global Initiative’s goals to improve childhood cancer survival and decrease disease burden and suffering.

There has not yet been a comprehensive systematic review and synthesis of extant literature characterizing the global impact of COVID-19 on pediatric oncology populations with respect to clinical outcomes and care delivery. As such, the primary objectives of this systematic review were to consolidate the literature with respect to these associations. Moreover, the literature was also reviewed to summarize recommendations and highlight priorities needed to remain on track to achieve WHO’s outlined goals for childhood cancer within the next decade.

## 2 Methodology

### 2.1 Search Strategy and Study Selection

Following PRISMA guidelines (Preferred Reporting Items for Systematic Review and Meta-Analyses), we systematically searched PubMed, Embase, CINAHL, and the African Medical Index using the following database-adapted search terms: “Pediatrics,” “COVID-19” and “Oncology”. The search terms were modified according to the specific vocabulary map of each database, and subsequently searched for papers from January 1, 2019 to November 3, 2021 without any restrictions (See **Appendix A**).

The removal of duplicates and initial screening was performed in Endnote (Clarivate Analytics, Philadelphia, PA, USA). After the removal of duplicate studies across all databases, article titles and abstracts were screened for relevance. Two reviewers (AM and ALM) independently reviewed the studies in accordance with pre-determined eligibility criteria. Articles without an abstract, or those which passed initial eligibility screening, were accessed in full-text for final inclusion/exclusion based on eligibility criteria. Consensus was reached in a follow-up discussion for papers whose eligibility was unclear.

We included any studies that reported an association between COVID-19 and cancer outcomes and/or cancer care delivery in pediatric populations (0-21 years). Data submitted as of November 3, 2021 were included in this report. All types of neoplasms and malignant cancers were included for analysis. Cancer care delivery was defined as any aspect of providing and distributing healthcare services to patient populations. All cancer outcomes (e.g., mortality, morbidity) and methods of cancer care delivery were included. Moreover, all age groups, sexes, ethnicities, and nationalities were included without any language restrictions. Articles were excluded if they: 1) were not available in full text and could not be accessed; 2) were mechanistic studies or preclinical studies; and/or 3) were duplicate studies. The systematic review was registered onto the International Prospective Register of Systematic Reviews (PROSPERO) at CRD42021266758.

### 2.2 Data Extraction

Data was extracted using a standard data extraction form. The following information was obtained: 1) Primary author & year; 2) Study design; 3) Country of Origin; 4) Study population; 4) Methodology; 5) Primary outcomes; and 6) Main findings.

### 2.3 Quality Assessment

The risk of bias of included studies was assessed using the GRADE Quality Assessment framework, a critical appraisal tool to rate the certainty of available evidence from “high” to “very low” (18).

## 3 Results

### 3.1 Search Results

**Figure 1:**
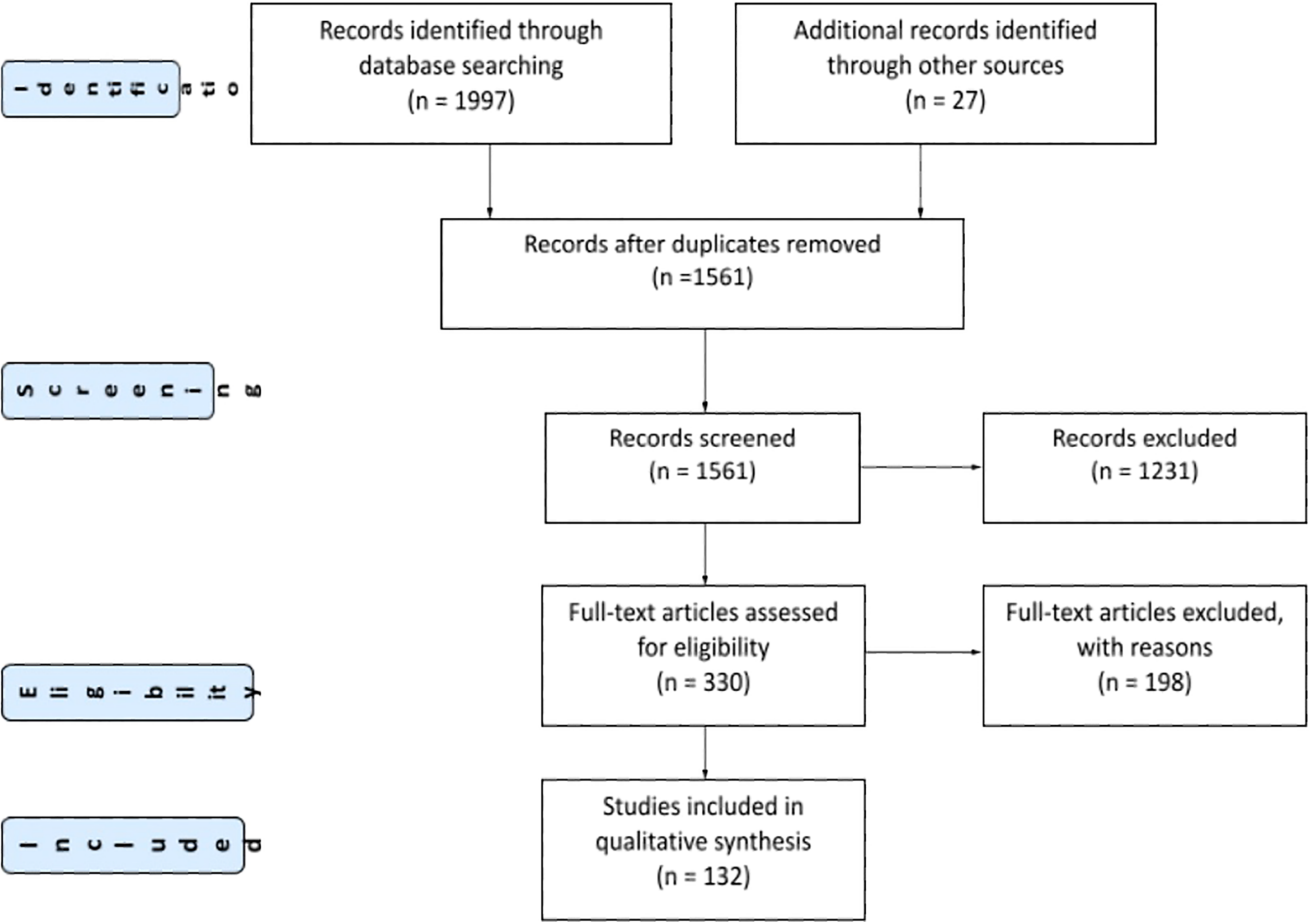
PRISMA Diagram.

The study selection process is depicted in the PRISMA flowchart (**Figure 1**). The systematic search from all databases yielded a total of 1561 records. After title and abstract review, 1231 records were excluded, and 330 full-text publications were assessed for eligibility. After full-text review, a further 198 studies were excluded. Additional studies were identified through a subsequent manual search of relevant papers. In total, 132 studies were included with 4398 patients, including 89 articles that examined clinical outcomes of COVID-19 in pediatric patients and 60 studies evaluating cancer care delivery during the pandemic, with some overlap (see **Appendix B**).

### 3.2 Clinical Outcomes of COVID-19 in Pediatric Patients With Cancer

We identified 89 studies that evaluated the clinical outcomes of COVID-19 in pediatric patients with cancer (see **Appendix B** **)**. From all high-income (HICs) and LMICs studied, there was an overall predominance of male pediatric oncology patients with COVID-19 (60.2% and 60.4%, respectively). Moreover, there was a predominance of pediatric hematological cancer patients with COVID-19 in each of the HICs and LMICs included in our review (63.7% and 61.8%, respectively). Three articles studied the clinical outcomes of COVID-19 in pediatric oncology patients globally (7, 19, 20). In a web survey by Hrusak et al. (2020) early in the pandemic, the authors found that amongst children receiving anticancer treatment globally (including 25 countries in Europe, Asia, Australia, and the Middle East), only 9 cases of COVID-19 infection were identified from over 200 patients tested, with 8/9 of patients presenting with an asymptomatic or mild course of the disease (19). A similar study by the American Society of Hematology noted that of 250 patients with blood cancers and COVID-19 from 74 sites across all continents, 20 (8%) were pediatric patients (<19 years old); 21% of patients had moderate or severe course of disease and the mortality rate was 11.1% (20).

More recently, Mukkada et al. (2021) conducted a global cohort study on pediatric oncology patients with COVID-19 (7). From 1500 submitted reports from 45 countries globally, the authors noted that 19.9% of patients had severe or critical infection and 50 deaths were attributed to COVID-19 infection (3.8%). A higher proportion of severe or critical illness was reported in LMICs when compared to HICs (OR: 5.8, [95% CI: 38–8·8]; p<0·0001). Interestingly, in univariate and multivariate logistic regressions, the authors noted that having a hematological malignancy and the presence of comorbidities were among factors significantly associated with severe or critical illness, alongside treatment intensity, older age (age 15-18), and country income group other than HIC (7).

#### 3.2.1 Clinical COVID-19 Outcomes in Children With Cancer Residing in HICs

The majority of studies were conducted in HICs (n=49), as defined by the World Bank criteria as of July 2021 (100), including Austria, Canada, France, Japan, Kuwait, Oman, and Poland, Saudi Arabia, Spain, Switzerland, United Arab Emirates, and the United Kingdom. Notably, the majority of studies were conducted in Italy and the United States (**Table 1**). Overall, 1518 patients in HICs were identified to have both a cancer diagnosis and COVID-19. ^1^ Of 49 studies that stratified cancer types, 928/1470 patients had hematological cancers (63.7%), while 542 patients had solid tumors (36.3%). Clinical symptoms and outcomes of all patients are characterized in **Table 1**. The majority of pediatric oncology patients with COVID-19 from HICs presented as asymptomatic (33.7%) or with primary manifestations of fever (36.1%) or respiratory symptoms (26.6%). One study did not stratify for patient clinical symptoms (**Table 3**) (23).

**Table 1:** Baseline characteristics of clinical outcomes for pediatric oncology children with COVID-19 in high-income countries (n = 49 studies).

Characteristics of included HIC studies	
*Countries*	*Number of studies, n*
Italy	6
United States	19
France	3
Poland	4
Saudi Arabia	2
Spain	5
Kuwait	1
Greece	2
Austria, Canada, Japan, Oman, United Arab Emirates, Switzerland, United Kingdom	7 (one case report or case series conducted in each country)
***Gender (applicable in 46/49 studies, 1500 patients)***	
Male	904/1500 (60.2%)
Female	596/1500 (39.7%)
***Type of malignancy (applicable in 45/49 studies, 1470 patients)***	
Hematological	928/1470 (63.7%)
Solid	542/1470 (36.3%)
Required critical care (i.e., ICU, mechanical ventilation)	116/1518(7.6%)
**Mortality rate (of 1518 patients)**	**28/1518 (1.8%)**

The bold values are the crude mortality rates.

From 1518 pediatric oncology patients in HICs who were infected with COVID-19, 28 patients died from COVID-19 related complications (crude mortality rate: 1.8%) and were often patients who presented with COVID-19 and refractory or metastatic cancer diagnoses or significant comorbidities. Fifteen of 31 patients were described in an observational study by Johnston et al. (2021) (24). The authors noted that of 917 pediatric oncology patients with COVID-19 in 34 US states, 10.9% of patients required respiratory support, 9.2% were admitted to the hospital, and 1.6% died due to the virus (15 patients, 60% of whom died due to the virus and 40% who died from a combination of cancer and SARS-CoV-2). The presence of comorbidities, such as obesity, hypertension, and asthma, was associated with an increased risk of hospitalization (RR = 1.3, 95% CI: 1.1-1.6) and ICU admission (aRR = 2.3, 9% CI: 1.5-3.6) (24). In another US retrospective cohort study by Kamdar et al. (2021), the authors found that 2/51 patients died from COVID-19; one patient had relapsed T-ALL while another had high-risk neuroblastoma. Both patients developed hypoxia progressive to respiratory failure and shock that was unresponsive to treatment with remdesivir and dextromethorphan (25). In a cross-sectional, cross-national study in Kuwait and Saudi Arabia, Alfraij et al. (2020) also described the case of one COVID-19 related death in a pediatric cancer patient; of 8 medical centers in Kuwait and Saudi Arabia, five pediatric cancer patients required ICU care for COVID-19 and one patient died. In univariate analysis, the presence of a comorbidity was associated with in-ICU mortality (HR=0.0001, 95% 0.0001-0.00016) (23).

The majority of studies, however, described positive outcomes for pediatric cancer patients with COVID-19 with asymptomatic presentations or mild or moderate disease course, and minimal changes to cancer care outcomes. Nine studies described patients who required ICU support due to deterioration but did not die due to the disease (26). Faura et al. (2020), for example, found that of 46 patients in a cohort study in Spain, the majority of patients presented with mild symptoms (51.1%) or were asymptomatic (25.5%); 11 patients presented with a severe illness course, 4 of whom required PICU care (8%) (27).

Notably, Ding et al. (2020) reported a case series of 5 patients, each of whom required emergency life-saving interventions shortly after presentation, including emergency intubation (n=4), resuscitation following cardiac arrest (n=2), and pericardiocentesis for tamponade (n=1). Two patients died within days of presentation. Interestingly, the authors noted that there was an increase in the percentage of patients requiring prolonged PICU care at cancer diagnosis during the pandemic. In April 2020, the authors noted that 75% of new leukemia/lymphoma patients diagnosed required PICU compared to the monthly average of 12% during 2018-19 (exact number of patients not reported) (28).

#### 3.2.2 Clinical Outcomes of COVID-19 in Children Residing in LMICs

Thirty-six studies (n=36) were conducted in low- and middle-income countries (LMICs), of which included Algeria, Brazil, China, Colombia, Egypt, India, Iran, Mexico, Pakistan, Peru, Russia, and Turkey (**Table 2**). A total of 873 patients from LMICs were studied^^2^^, from which the majority had hematological malignancies (535/865, 61.8%) while a minority were diagnosed with solid tumors (330/865, 38.1%). The majority of pediatric oncology patients were either asymptomatic for COVID-19 (22.0%) or had primary symptoms of fever (51.4%), cough (23.6%), or respiratory symptoms (11.7%). Patient clinical outcomes are characterized in **Table 3**.

**Table 2:** Baseline characteristics of clinical outcomes for pediatric oncology children with COVID-19 in low-and-middle income countries (n = 36 studies).

Characteristics of included LMIC studies	
*Characteristics*	*Number of studies, n*
Algeria	1
Brazil	2
China	3
Colombia	1
Egypt	3
India	12
Iran	2
Mexico	4
Pakistan	2
Peru	1
Russia	2
Turkey	3
***Gender (applicable in 33/36 studies, 820patients)***	
Male	496/820 (60.4%)
Female	324/820 (39.5%)
***Type of malignancy (applicable in 35/36 studies, 865 patients)***	
Hematological	535/865(61.8%)
Solid	330/865 (38.1%)
Required critical care (i.e., ICU, mechanical ventilation)	98/830(11.8%)
**Mortality rate**	**70/873 (8.0%)**

The bold values are the crude mortality rates.

**Table 3:** Primary symptoms of COVID-19 in pediatric patients with active cancer in HIC and LMICs.

Symptoms of COVID-19 in pediatric patients with active cancer	HICs(N = 1518 patients)		LMICs (N = 830 patients)	
	Number of patients, N	Percentage of patients	Number of patients, N	Percentage of patients, %
Asymptomatic	511	33.7	149	22.0
Fever	548	36.1	427	51.4
Cough	98	6.4	196	23.6
Respiratory symptoms (e.g., dyspnea, respiratory distress)	450	29.6	97	11.7
Fatigue and malaise	25	1.6	0	0
Flu-like symptoms (e.g., rhinitis, rhinorrhea, sore throat)	27	1.8	98	11.1
GI symptoms (e.g., diarrhea, vomiting, gastroenteritis)	22	1.4	88	10.6

2 studies did not stratify for patient clinical outcomes (21, 22).

From 873 pediatric oncology patients in LMICs, 70 patients died due to COVID-19 related complications (mortality: 8.0%) (see **Appendix B** **).** Seven of 70 patients were reported in a Mexican retrospective study of patients with acute leukemia (mortality rate: 46.7%, 7/15 patients) (29). The authors noted that the risk of death in children with leukemia and COVID-19 was associated with increased liver enzymes, respiratory distress, and the need for mechanical ventilation, amongst other variables (29). Montoya et al. (2020) also described a report of 7 deaths among 60 patients with COVID-19 and a cancer diagnosis in Peru (mortality rate: 10%; both hematological and solid tumors considered); 4 patients died from complications and progressive disease that the authors noted were not related to COVID-19 while 3 patients had severe pneumonia and deteriorated despite ICU care (n=1) or unavailable ICU care (30). The authors noted that patients under non-curative treatment (i.e., palliative care) had a higher risk of death due to COVID-19 than those under curative treatment (30).

Three patients deaths were reported in India wherein all patients had refractory hematological cancers (leukemia, lymphomas) (21). Ganguly et al. (2020) highlighted that of the cases in their care, one patient committed suicide in fear of contracting COVID-19, underlining the excess of fear, stress, and stigma in the community (21). Ten patient deaths were reported in Egypt; Hammad et al. (2021) found that from 76 pediatric oncology patients admitted to the Children’s Cancer Hospital from May-November 2020, 10 patients presented with severe or critical disease and died (31). Corso et al. (2021) also noted that from 179 patients they surveyed, the severity of presentation at diagnosis was significantly associated with fatal outcomes (p<0.001); of 22 deaths, 10 patients had leukemia, 2 had lymphoma, and 8 had solid tumors, while 2 patients had other cancer diagnoses (32).

Several studies found that pediatric oncology patients required intensive care support due to severe or critical disease. Notably, Totadri et al. (2021) noted that of 2115 children under 15 years old tested for COVID-19 in southern India, 37 patients were positive and had cancer diagnoses. The most common underlying malignancies were ALL (n=16) and Ewing sarcoma (n=7) (33). While the majority of patients were asymptomatic or had mild/moderate courses of disease (n=23, 62%), 12 patients presented with severe courses of hypoxia, tachypnea, and shock. Two patients had critical disease and required mechanical ventilation, experiencing shock requiring vasoactive support, andhad organ dysfunction, and/or coagulopathy (33). The authors postulated that contributing factors to the illness were oncological emergencies (n=2), dengue co-infection (n=4), and inguinal bacterial abscess (n=1). However, all patients were discharged in a stable condition (33).

### 3.3 Healthcare Delivery for Pediatric Patients With Cancer During the Pandemic

We identified 60 studies that reported changes to healthcare delivery for pediatric oncology patients during the COVID-19 pandemic (**Appendix B**).

#### 3.3.1 Healthcare Delivery for Pediatric Oncology Patients With COVID-19 in HICs

Thirty-five articles were conducted in HICs, which included France (n=2), Germany (n=2), Greece (n=1), Israel (n=1), Italy (n=11), Kuwait (n=1), the Netherlands (n=1), Norway (n=1), Saudi Arabia (n=2), Spain (n=1), the United Kingdom (n=2), and the United States (n=10) ^3^ . The majority of studies outlining impacts to care delivery were reported in conjunction with clinical outcomes.

In general, many studies reported delaying oncology treatment and chemotherapy for pediatric patients who had concurrent COVID-19 infection; oncological therapies were often recommended after the infection cleared. Ferrari et al. (2020), for example, noted that of 21 patients in treatment in onco-hematology centers in Lombardia, Italy, 38% of patients had altered their cancer treatment due to the infection, including delaying chemotherapy, postponing transplant surgeries, or reducing drug dosages. While these patients were not followed-up, the authors noted that due to the aggressive nature of pediatric tumors, postponing therapy may have jeopardized the therapy’s efficacy and reduced patient cure rates (34). Similarly, in a retrospective cohort study by Madhusoodan et al. (2020), the researchers noted that of 13 pediatric oncology institutions in the New York/New Jersey region, interruptions to chemotherapy treatment were reported in 67% of all patients (66/98). Delays in cancer-directed surgery or radiation were reported in 6.1% (6/98) of SARS-CoV-2 patients regardless of COVID-19 disease severity (35). Similar interruptions to oncology care were reported in case reports and cohort studies of pediatric oncology patients in other regions of the US (36, 37) Saudi Arabia^,37^ Kuwait (38), Italy (39, 40), and France (41).

Rouger-Gaudichon et al. (2021) noted that per respondents to a questionnaire distributed to 31 pediatric oncology centers in France, 17.9% declared that medical care quality was degraded for all pediatric oncology patients due to the pandemic, regardless of whether they had contracted the virus (42). Twenty centers (71.4%) noted that supportive care quality was also decreased; social and psychological follow-up was conducted virtually in 18 of 20 centers (42). Moreover, many studies did not report fatal outcomes in pediatric oncology patients due to the treatment delay required due to the COVID-19 infection; however, follow-up times were short and thus the impact of these treatment delays on overall survival cannot be definitively determined. Several studies noted that with the clearance of the virus (often, without any COVID-19-specific treatment) and/or for patients who were clinically stable, patients were able to resume chemotherapy treatment as per protocol (34, 37, 43, 44).

Reductions in hospital services due to COVID-19 were not unique to HICs in Europe. In a cross-sectional observational study of 204 children being treated for leukemia/lymphoma (60.4%) or solid tumors (39.6%) in Saudi Arabia, Alshaharni et al. (2020) noted similar treatment delays and reductions in services due to COVID-19. The most common reasons for treatment delays were limited hospital capacity due to social distancing guidelines, hospital cancellations, city lockdown and curfew, and swab requirements prior to admission (45). 30.8% of patients also reported non-availability of adequate PPE, lack of cancer support, and shortage of medications due to the pandemic (45). The COVID-19 pandemic has added additional burden to families with children being treated for cancer. In a cross-sectional UK study by Darlington et al. (2020), 69.6% of parents of pediatric oncology patients (n=171) reported feelings of anxiety and feeling unsafe in hospital settings due the pandemic (46). However, these reported worries did not significantly lead to parents stopping or reducing chemotherapy for the child; indeed, only 2% of 171 parents opted for this route (46).

In several instances, hospitals were able to restructure services to better provide care for patients, including guidelines for social distancing (14, 47–50). For example, Vagelli et al. (2020) noted that in a tertiary center in Italy, the hospital team split the hospital into two sections, with one section to manage patients at risk or with suspected/confirmed COVID-19; pediatric oncology patients with COVID-19 were admitted and isolated in the COVID-19 unit if they needed continuous radiotherapy or had complex chemotherapy regimens. Specific routes for patients and healthcare workers were also developed to avoid cross-contamination between patients and potential sources (48). Similar strategies were also employed by the Italian Pediatric Oncology and Hematology Association centers, who noted that in order to control the spread of the virus, 24 centers (67%) redesigned facility common areas and made structural changes to follow social distancing guidelines (49).

Altogether, many HICs reported less pediatric oncology diagnoses when compared to before the pandemic, especially in relation to solid tumour diagnoses (**Appendix B**). Chiaravelli et al. (2020) reported only 16 newly diagnosed cases of pediatric solid tumors from March-May 2020 at the Pediatric Oncology Unit of the Istituto Nazionale Tumori, a referral center for pediatric solid tumors for all of Italy. During the same periods in between 2017-2019, the hospital registered 34, 35, and 36 cases, signifying an absolute decrease of 45.7% cases (p=0.0416). In the eight subsequent weeks following the Italian lockdown (May-June 2020), however, the number of cases registered was in line with previous years, without any rebound (51). Similarly, Offenbacher et al. (2021) noted a statistically significant decrease in solid tumor diagnoses during the pandemic- from an average of 13.6 cases (7.9-19.3) from 2015-2019 to only 4 new cases during 2020 (52). This delay in diagnosis was not reflected in leukemia patients (mean of 5.2 [3.2-6.8] cases in 2015-2019 and 4 cases during 2020) (52).

Contrarily, Ding et al. (2020) noted that despite a historic mean of 2.96 days between new leukemia patients, the Children’s Hospital of Philadelphia did not see any new patients with a leukemia diagnosis for 35 days (March 2-April 6, 2020; the longest gap from 2015-2019 was 18 days) (28). Subsequently, and on consecutive days, two patients presented to the local hospital PICU with an as yet undiagnosed ALL and cardiac arrest. Jarvis et al. (2021) also noted that while the number of solid tumours diagnosed during COVID-19 in the south-eastern region of Norway remained stable from before and during COVID-19, there was a marked reduction in ALL cases (53). During 2017-2019, there was an average of 2.03 cases/month (95% CI 1.63-2.43)- this in stark contrast to the zero diagnoses of new ALL cases made during the first six months of 2020, a probability of which the authors note is less than 0.001 (95% CI 0.00006-0.001) (53).

#### 3.3.2 Healthcare Delivery for Pediatric Oncology Patients With COVID-19 in LMICs

Twenty-four studies were conducted in LMICs, including Armenia (n=1), China (n=1), Mexico (n=1), Turkey (n=2), India (n=10), Iran (n=2), and Pakistan (n=4) (**Appendix B**). Similar to HICs, studies conducted in LMICs reported delays to chemotherapy (33, 54–57), decrease in surgery rates (55, 58), and decline in the number of new diagnoses and supportive care services offered (i.e., palliative care, psychologist, social welfare services, residential supports) (59, 60). One comprehensive retrospective chart review by Kutluk and colleagues (2021) noted that in a major Turkish pediatric oncology department, outpatient and inpatient visits were reduced by 29.6% (p<0.0001) from March-October 2020 as compared to an analogous time period before the pandemic (61). Similarly, the mean number of inpatients were reduced by 25.5% (p<0.00001); from 23.8 patients/day to an average of 17.7 patients/day during the pandemic period (61). The number of chemotherapies (14.19/day vs 17.92; p<0.0001), radiotherapies (6.04/day vs 6.79, p=0.035), surgical procedures (1.59/day vs 2.36, p=0.000015), and imaging studies (12.46/day vs 19.62, p<0.0001) conducted were also reduced as compared to the control period (61).

Case series and surveys conducted in Mexico and Armenia, respectively, noted that the main challenges during the COVID-19 pandemic were staff shortages (reduced to 50% in a single center in Mexico City) due to positive cases and direct contact with infected staff (62, 63). At one hematology center in Armenia, researchers noted supply chain disruptions and delays (e.g., chemotherapy infusions postponed) as well as increases in the price of certain drugs and supplies (e.g., the price of PPE increased by more than 10 times) (62).

The reorganization of hospital services were reported to have mixed results (21, 59, 64). Ganguly et al. (2021), for example, noted that in a tertiary government healthcare center in India, conversion to telemedicine greatly benefitted their patients; in the period between April-July 2020, the number of teleconsultations rose exponentially (32 in April to 197 in July, 2020). Chemotherapy plans and prescriptions issues were also managed through email and were especially helpful for patients who lived far from the medical center (21). It was not specified, however, how patients who did not have access to internet or electricity fared with these changes. Sajid et al. (2021) also noted a reorganization of pandemic services in a single healthcare institution in Pakistan; however, to more negative results (64). The authors noted that due to shared inpatient space, any child suspected of being exposed to a healthcare provider with suspected COVID-19 would lead to a cascade of exposed children with multiple children requiring isolation and testing (64). Testing every febrile neutropenic patient with COVID-19 led to undue healthcare expenditures for most self-paying families. As well, many families did not have access to smartphones or the internet making teleconsultation and receiving prescriptions challenging, especially for patients who lived in rural areas and did not have other healthcare access or transport services (64). In another study, Bansal et al. (2021) noted that of the 326 children with retinoblastoma seen nationwide in India, distance was a major deterrent for follow-up and receiving care, and which severely impacted follow-up care for families with middle and lower socioeconomic status (43% and 41% respectively). A mean therapy delay of 45.8 ± 24.3 weeks was reported and the authors noted that disease activity worsened in 60% of active cases (65).

Notably, more severe outcomes were reported in LMICs than in studies conducted in HICs. From a study that gathered survey data across 15 African francophone countries, Traore et al. (2021) reported a severe shortage of hospital equipment, including 50% of participants reporting a shortage of blood supplies, lack of PPE in 80% (20/25) of centres interviewed, and lack of COVID-19 care units in the majority of pediatric oncology centres (66). Indeed, only 35% of facilities had adapted their services for COVID-19, including isolation for patients suspected of having COVID-19 (17%) and only 4% of centres had a special hospital circulation route for patients with cancer to protect them from coming into contact with COVID-19 patients. Moreover, 60% of centres reduced their activities because of staff shortages and postponed surveillance consultations (66). In a similar study of 20 Latin American countries by Vasquez et al. (2021), pediatric onco-hematologists noted indefinite postponement of surveillance consultations (89% of participants), cancer surgeries (45%), radiotherapy schedules (33%), and outpatient consultations (26%) (13). A shortage of healthcare supplies was noted by 79% of participants (e.g., blood products, pathology samples, PPE), which was more frequent in countries with a health-care expenditure of less than 7% of GDP, high COVID-19 incidence rates, and with more stringent travel restrictions (13). The Pediatric Oncology East and Mediterranean group found analogous results when surveying 34 centres from the Middle East, North Africa, and West Asian regions (**Appendix B**). In the recent global study by Mukkada et al. (2021), the authors also noted that chemotherapy was withheld or modified in 44.6% and 55.8% of patients receiving active therapy, respectively. Interestingly, in multivariable analysis, treatment modifications were noted to be most significantly associated with income group, specifically for UMICs [OR=0.5 (95% CI 0.3-0.7)] (7).

Graetz et al. (2021) distributed a survey to 79 countries and 213 institutions from all WHO regions and found that while pediatric cancer care has been affected globally, this effect was more pronounced in LMICs. Thirteen of 15 hospitals that completely closed their pediatric oncology units during the pandemic were located in LMICs, and LMICs were more likely to report decreases in the financial support they received from their respective governments during this time (p=0.0004). As a consequence, the long-standing issue of treatment abandonment in LMICs was thus further compounded by the pandemic, and much more so than for HICs (p<0.0001). Moreover, LMIC institutions also reported additional barriers to life-saving interventions (p=0.019), more severe complications (p=0.0059), and deaths (p<0.0001) due to COVID-19 (67).

## 4 Discussion

### 4.1 Clinical Outcomes

Overall, the majority of studies reported patients with cancer and COVID-19 presenting with symptoms of fever (HICs: 36.1%, LMICs: 51.4%) and/or respiratory symptoms (HICs: 29.6%, LMICs: 11.7%). Interestingly, moderate to large proportions of patients with cancer and COVID-19 were reported as asymptomatic in both HICs (33.7%) and LMICs (22.0%). These findings are similar to those reported in other analyses of COVID-19 in pediatric patients without cancer, further confirming that children infected with COVID-19 have a generally milder disease course and better prognosis than adults, regardless of whether the patient is from a HIC or LMIC (68–73). In a meta-analysis by Dorantes-Acosta et al. (2020), for example, the authors found that amongst eight studies of pediatric oncology patients with COVID-19 in France, Italy, the US, Spain, and Mexico, a 100% survival rate was reported. Most COVID-19 cases in this review were in patients with leukemia, and fever was the most common symptom requiring admission in children, as was also found within our study (74).

These results are interesting insofar as one of the primary consequences of cancer therapies is immunosuppression in both pediatric and adult patient populations. Ruggiero et al. (2020) note that children receiving immunosuppressive chemotherapy have both qualitative and quantitative abnormalities in T cell function and immunoglobulin levels that persist for months after the completion of therapy (75). Indeed, in adult cancer patients, COVID-19 has posed a substantial risk for morbidity and mortality (4, 76, 77). In a recent systematic review, El-Gohary et al. (2020) found that adult cancer patients with COVID-19 had a higher risk of severe and critical disease, ICU admission, mechanical ventilation, and higher mortality rates as compared to noncancer patients, and had higher risk of COVID-19-related complications (77). Our review and analysis thus suggests a different course for COVID-19 in pediatric oncology patients.

Overall, there was predominance of male pediatric oncology patients with COVID-19 (HICs: 60.2%, LMICs: 60.4%). This male predominance has been observed for COVID-19 severity in adults across multiple studies and aligns with the finding that males have increased susceptibility to infectious diseases of various kinds (78, 79). Moreover, as also described in adults (80, 81), there was a predominance of pediatric hematological cancer patients with COVID-19 in each of the HICs and LMICs included in our review (63.7% and 61.8%, respectively). It is also interesting to note that, in both HICs and LMICs reported in this review, the majority of patients who died from COVID-19 were those patients with progressive or refractory cancers (52, 82) or who had significant comorbidities additional to their cancer diagnosis (23). Altogether, these results and previous findings speak to altered mechanisms for COVID-19 infection in pediatric populations leading to a milder course of the infection (83).

Moreover, while the majority of patients in both HICs and LMICs were generally noted to have a good prognosis (survival of 98.2% and 92.0%, respectively), there was an obvious discrepancy in this data. Indeed, while 28/1518 pediatric patients with cancer died of COVID-19-related causes in HICs, this proportion increased to 70/874 patients in LMICs. This is in alignment with previous research demonstrating higher mortality rates in pediatric patients with COVID-19 in LMICs as compared to HICs. In a recent systematic review, Kitano et al. (2021) reported that pediatric deaths/million children and case fatality rates were significantly higher in LMICs than in HICs (2.77 in LMICs vs 1.32 in HICs, p<0.001; and 0.24% vs 0.01%, p<0.001, respectively) (84). Higher mortality rates for pediatric patients in LMICs have not only been reported for COVID-19, but also many other infections and illnesses including respiratory infections (85), pertussis (86), and sepsis (87), amongst others.

It appears highly likely that these discrepancies in mortality data are due to differences in COVID-19 testing, presentation, and level of care influencing symptom profile and prognosis depending on the income level of the country. Moreover, underlying socioeconomic disparities likely add data discrepancies, with decreased access to healthcare facilities in the case of COVID-19 infection and decreased supportive care mechanisms, amongst other barriers (64, 65). In a review conducted in September 2020, Zar et al. (2020) also noted that while the direct effects of COVID-19 in LMICs are less of a concern in children who remain largely asymptomatic or develop mild illness as in HICs, indirect effects in LMICs may be more worrisome, and impact prognosis (88). These include difficulties of maintaining regular childhood preventive and curative services as healthcare resources are constrained, diversion of resources from child health to address pandemic health in adults, and the timely identification of affected individuals with subsequent self-isolation (88).

### 4.2 Healthcare Delivery

From the 60 studies that reported upon the impact of COVID-19 on healthcare delivery in HICs and LMICs, there were delays and interruptions in cancer therapies as well as delays in diagnosis. Ferrari et al. (2020), for example, noted the postponement of transplant surgeries, delays in chemotherapy, and reductions in drug dosages in a tertiary center in Italy (34). In a French study by Rouger-Gaudichon et al. (2020), the authors noted that, from a survey of medical centers that took care of COVID-19 patients, many centers noted a degradation of medical care quality in pediatric oncology including a decrease in supportive care quality, regardless of whether patients had contracted the virus (41).

There is a mixed picture regarding how COVID-19 has impacted delays to diagnosis for cancer. While some studies reported that delays in diagnosis were potentially occurring for some cancers, there is insufficient data to indicate what impact COVID-19 has on delayed diagnoses or data to report on which particular category of cancers are experiencing greater diagnostic delays. This is in line with previous research demonstrating national and international variability regarding diagnostic delays for childhood cancers, often dependent on type or ways of measuring these delays. These delays are postulated to be due to increased fear of catching COVID-19 in the healthcare system, lockdowns, restricted public transportation and access to the healthcare centers, and hospital prioritization for patients with COVID-19 (34, 51, 52, 89, 90).

Healthcare delays and disruptions have been particularly increased for pediatric oncology patients in LMICs. Staff shortages were described primarily in LMICs, with supply chain disruptions, and increase in prices of chemotherapeutic drugs and PPE (62). These results are well captured by Graetz et al. (2021) who described pronounced health service disruptions to childhood cancer care in LMICs, with a greater number of hospital closures, decrease in financial support from the government, and limited access to cancer therapies (67). Overall, these results indicate that care delivery in COVID-19 is reflective of healthcare systems around the world. Before the pandemic, many LMIC healthcare systems had already been strained with decreased resources and access for care for children with cancer (91, 92). In the time of COVID-19, these institutions have noted greater staff and supply shortages, treatment modification, and lack of supportive care (58, 64, 67).

### 4.3 Limitations

There are several limitations of this study. First, while this study is a systematic review on the impact of COVID-19 on global pediatric cancer outcomes and care delivery, it is not a meta-analysis. Indeed, the methodological heterogeneity amongst reported studies (e.g., due to patient population, types of tumors studied, clinical outcomes measured) limited our ability to quantitatively assess data between cancer diagnoses and between HICs/LMICs. Moreover, the limited follow-up time for many of the reported studies rendered us unable to make concrete conclusions about mortality rates for pediatric oncology patients affected by COVID-19. It is also important to note that, while the majority of studies confirmed COVID-19 diagnosis in children through RT-PCR, a small subset of studies did not specifically report how the diagnosis of COVID-19 was made (i.e., 7 studies) (21, 24, 38, 93–96); we have included these articles for completeness. Further, several studies included in this review were case reports or cohort/cross-sectional studies written up as letters to the editor. As such, our ability to complete a risk of bias assessment for studies varied (see **Appendix B** for risk of bias assessment).

Additionally, while we aimed to consolidate information from all countries, there was a discrepancy between the number of studies reported in HICs and LMICs. For example, there were only 36 (n=873 patients) studies that reported clinical outcomes in LMICs while 49 studies (n=1518 patients) were reported in HICs. As well, some countries had a preponderance of studies; in HICs, for example, the majority of studies were conducted in the United States (19/49). In LMICs, the majority of studies were reported in India (12/36). These discrepancies skewed data and impacted the analyses that we are able to make between countries. As well, while this paper presents an overview of the similarities and differences in clinical outcomes and care delivery between World Bank economies, it is difficult to compare outcomes between countries without also addressing the politics, economics, and health policies of each specific country. Future reviews should aim to incorporate these factors to make contextual conclusions and COVID-19 recommendations.

### 4.4 Conclusions and Avenues for Future Research

In the early months of the pandemic, Sullivan et al. (2020) created rapid response recommendations for children with cancer globally, including recommendations for patients with ALL, Burkitt’s lymphoma, and retinoblastoma, amongst others, as well as recommendations on how to effectively adopt and distribute treatment modalities including radiotherapy, surgery, nursing, and palliative care (44). Pritchard-Jones et al. (2021) also recommended ongoing psychosocial support for families, maintaining consistent and timely cancer services, using collaborative data registries such as the Global COVID-19 Registry to gain knowledge regarding COVID-19 in child cancer patients, and an early return to surgery and radiotherapy treatment (97). Recently, in a large US cohort study, Martin et al. (2021) observed that there were differences in demographic characteristics and pre-existing comorbidities between severity subgroups wherein marginalized populations (e.g., Black/African American race), being younger than 12 years, and having a pediatric complex chronic condition were predictive of peak disease severity (98). These results may suggest that early identification of children likely to progress to severe disease could be achieved using key data elements, and may help in the treatment of pediatric populations. As also noted by Graetz et al. (2021), teamwork, flexible and coordinated approaches to care, among-institution collaboration, partnerships with government, private, and non-profit organizations are key to fostering healthcare resilience (67), and should be prioritized as we remain in the depths of this pandemic and as we move beyond it.

This was the first study that aimed to comprehensively describe all published studies globally on the clinical outcomes and impacts to healthcare delivery in pediatric oncology patients with COVID-19, while stratifying for economies. Future studies should aim to assess the clinical impacts of COVID-19 in pediatric oncology amongst larger patient cohorts, and take into account vaccination and COVID-19 variants. Further, there need to be consolidated outcome measures that allow for evaluation of cancer care between patient cohorts both intra- and inter-nationally (98). With the delta and omicron variants, and future potential variants of COVID-19, it will also be imperative to determine how new strains of the virus impact clinical outcomes and access to healthcare for pediatric cancer patients globally.

In conclusion, COVID-19 has impacted pediatric oncology patients around the world, both in terms of clinical outcomes and care delivery. In order to meet the WHO Global Initiative for Childhood Cancer goals by 2030, it will be imperative to apply consolidated recommendations to diagnose, treat, and accommodate children with cancer during the pandemic and beyond.

## Data Availability Statement

The original contributions presented in the study are included in the article/**Supplementary Material**. Further inquiries can be directed to the corresponding author.

## Author Contributions

AM and ALM contributed to the overall design, article selection, review, and manuscript preparation. TW contributed to data extraction and review. BG contributed to data extraction and review. CL contributed to article identification, analysis and interpretation of data, critical revisions and manuscript review. RA contributed to article identification, analysis and interpretation of data, critical revisions and manuscript review. All authors contributed to the article and approved the submitted version.

## Conflict of Interest

The authors declare that the research was conducted in the absence of any commercial or financial relationships that could be construed as a potential conflict of interest.

## Publisher’s Note

All claims expressed in this article are solely those of the authors and do not necessarily represent those of their affiliated organizations, or those of the publisher, the editors and the reviewers. Any product that may be evaluated in this article, or claim that may be made by its manufacturer, is not guaranteed or endorsed by the publisher.

## Footnotes

1 Note that this statistic excludes multinational studies, which were mentioned earlier in *Section 3.2*.

2 This statistic excludes multinational studies, which were mentioned earlier in *Section 3.2*.

3 These numbers did not include multinational studies.
