# Supplementary material for: The Global Impact of COVID-19 on Childhood Cancer Outcomes and Care Delivery - A Systematic Review
Source: Front Oncol. 2022 Apr 7;12:869752. doi: 10.3389/fonc.2022.869752 (PMC9023072; doi:10.3389/fonc.2022.869752)

**Appendix A**

Database: **OVID Medline: Epub Ahead of Print, In-Process & Other Non-indexed Citations, OVID MEDLINE Daily and OVID MEDLINE** (1947-Present)
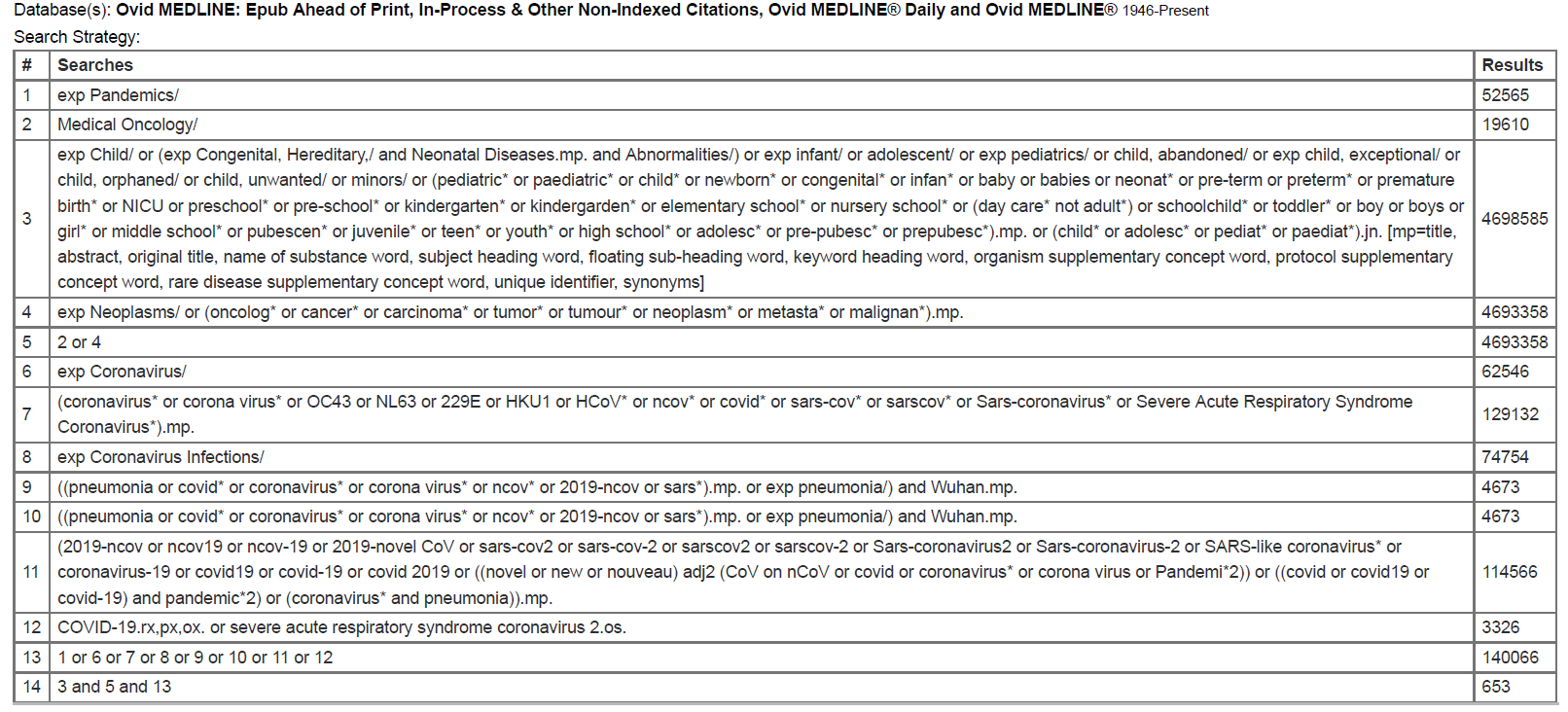


Database: **Embase Classic, Embase (**1947-2021)
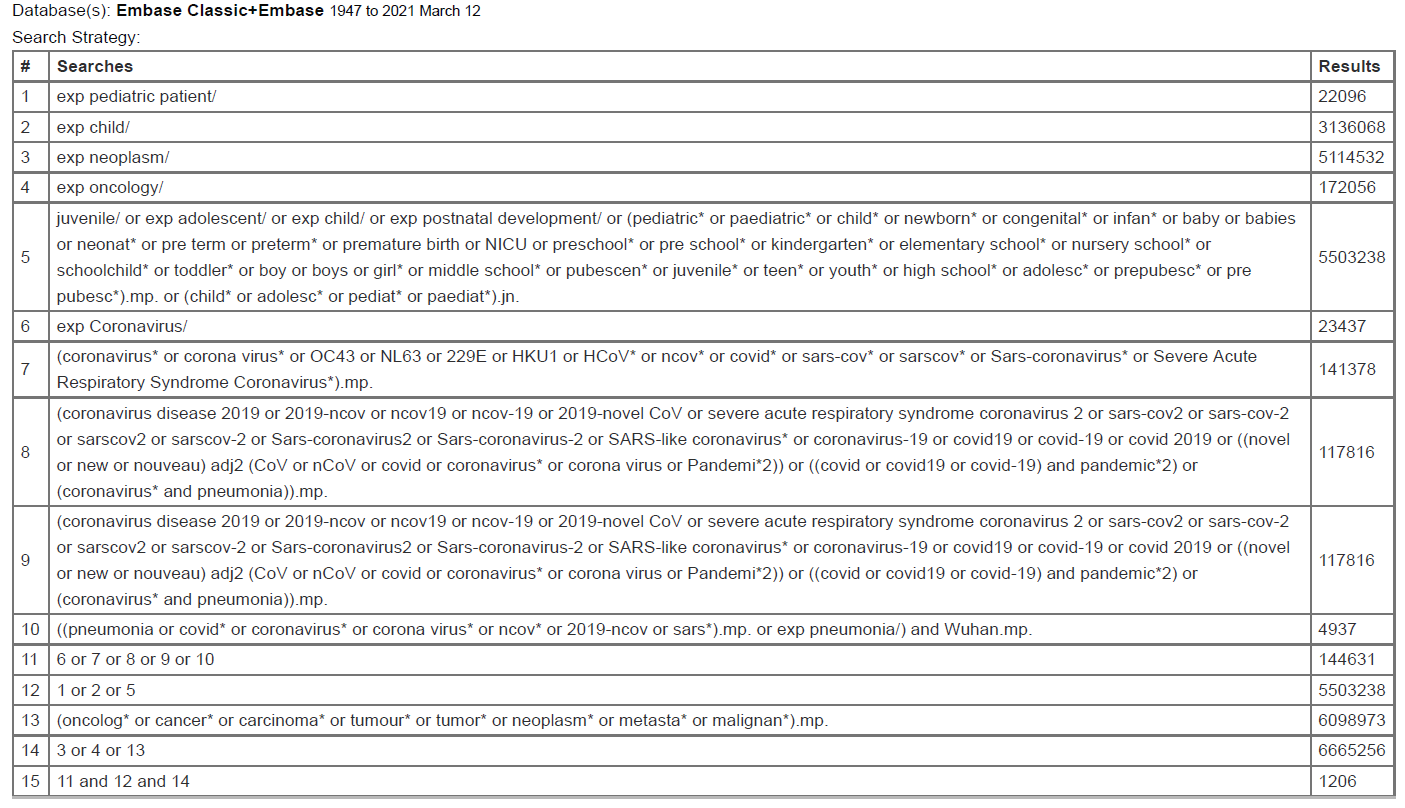


Database: **EBSCO CINAHL (**1947-Present)
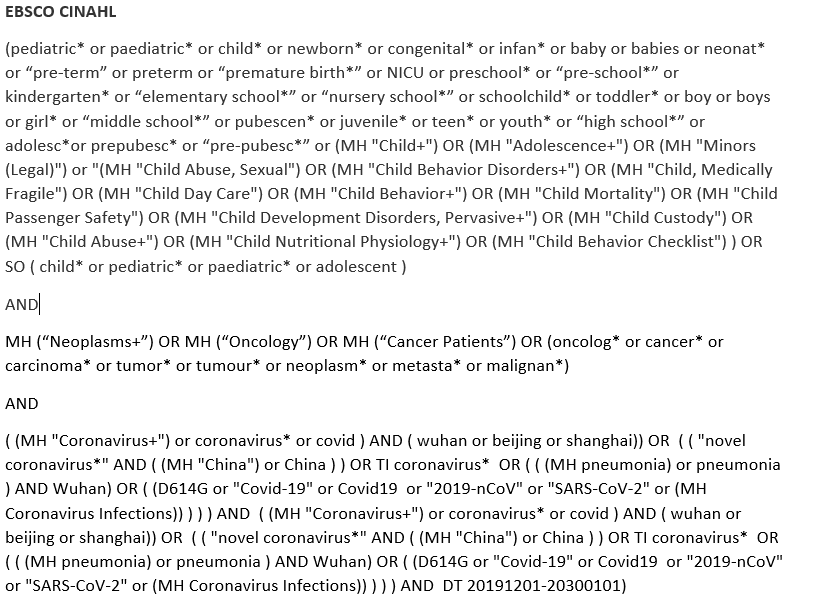

Supplement: Supplementary file 1 [file DataSheet_1.docx]
